# An RNA nanoparticle vaccine against Zika virus elicits antibody and CD8+ T cell responses in a mouse model

**DOI:** 10.1038/s41598-017-00193-w

**Published:** 2017-03-21

**Authors:** Jasdave S. Chahal, Tao Fang, Andrew W. Woodham, Omar F. Khan, Jingjing Ling, Daniel G. Anderson, Hidde L. Ploegh

**Affiliations:** 1 Whitehead Institute for Biomedical Research, 9 Cambridge Center, Cambridge, MA 02142 USA; 2 The Koch Institute for Integrative Cancer Research, Massachusetts Institute of Technology, Cambridge, MA 02142 USA; 3 Program in Cellular and Molecular Medicine, Boston Children’s Hospital, Boston, MA 02115 USA

## Abstract

The Zika virus (ZIKV) outbreak in the Americas and South Pacific poses a significant burden on human health because of ZIKV’s neurotropic effects in the course of fetal development. Vaccine candidates against ZIKV are coming online, but immunological tools to study anti-ZIKV responses in preclinical models, particularly T cell responses, remain sparse. We deployed RNA nanoparticle technology to create a vaccine candidate that elicited ZIKV E protein-specific IgG responses in C57BL/6 mice as assayed by ELISA. Using this tool, we identified a unique H-2D^b^-restricted epitope to which there was a CD8^+^ T cell response in mice immunized with our modified dendrimer-based RNA nanoparticle vaccine. These results demonstrate that this approach can be used to evaluate new candidate antigens and identify immune correlates without the use of live virus.

## Introduction

The ongoing outbreak of Zika virus (ZIKV), a flavivirus, in Latin America and the South Pacific is associated with an increased incidence of neurological complications, including Guillain-Barré syndrome^1, 2^ and fetal abnormalities, including spontaneous abortion, microencephaly, and placental insufficiency^3, 4^. At present there are no approved vaccines or specific treatments for ZIKV infection. In February 2016, the World Health Organization declared the Zika outbreak a Public Health Emergency of International Concern. Hundreds of cases have already been reported in the United States, attributed to acquisition by travel to affected areas. Local transmission is now prevalent in US Territories, particularly in Puerto Rico^5^. Mosquito-borne and sexual transmission on mainland US soil led to unprecedented warnings by the CDC for pregnant women and their sexual partners^6, 7^. The expanding habitat of the *Aedes* mosquito species that serves as a vector for ZIKV could spread this epidemic even further. Vaccine development efforts to date have yielded DNA-based candidates, one of which has entered a clinical trial^8, 9^. However, deployment of DNA-based immunoprophylactics requires electroporation or jet-injection systems^9, 10^. This makes administration of the lead vaccine candidate a challenge in most of the seriously affected regions. Therefore, developing new analytical tools that can accelerate further vaccine candidate research is of the utmost importance.

Zika viruses are broadly classified into Asian, East African, and West African lineages^11^. The current outbreak has been attributed to the Asian genotype^12–14^. The causative Western Hemisphere strains share a high degree of nucleotide identity within the clade (>99%). The long-studied prototypical ZIKV reference strain MR 766 was first isolated in Uganda in 1947, and is a member of the East African cluster. It is less homologous (~89%) to the Western Hemisphere strains. The frequent cross-reactivity among Flavivirus species complicates serological detection of ZIKV infection or virus-specific antibodies, making studies of ZIKV-specific humoral immunity challenging^15–19^. Susceptible adult animal models of ZIKV pathogenesis that recapitulate neurotropic disease exploit IFN receptor gene knockouts/blockade in C57BL/6 and other mouse strains carrying the MHC H-2^b^ haplotype^20–22^. Other IFN-deficient mouse strains are similarly susceptible to ZIKV infection^21^.

The geographic co-distribution and co-circulation of many serologically similar classes of arboviruses^23^ pose a hurdle to the characterization of ZIKV-specific immune responses. Commercially available enzyme-linked immunosorbent assay (ELISA) kits that detect anti-flaviviral antibodies suffer from cross-reactivity to other Flavivirus strains. This confounds the study of multivalent flaviviral vaccines.

In the C57BL/6 model of ZIKV infection, CD4^+^ T cell depletion does not abrogate protective efficacy of gene-based vaccines^24^. Therefore, cytotoxic CD8^+^ T cell responses likely play a role in protection against ZIKV infection, as for other flaviviruses^25–27^. MHC-restricted ZIKV epitopes remain to be defined. Even a single-peptide MHC class I determinant would aid current vaccine development efforts, as it would provide a parameter to screen vaccine performance in mice.

We previously developed a modified dendrimer nanoparticle (MDNP)-based RNA replicon vaccine platform that provides single-dose protection in mouse models of lethal Influenza, Ebola, and *Toxoplasma gondii* challenges^28^, and in the current study applied it to ZIKV. The vaccine induced detectable anti-ZIKV IgG responses in C57BL/6 mice. Analyses of the cellular response to the vaccine revealed an immunodominant H-2D^b^-restricted epitope derived from the ZIKV envelope (E) protein. By means of T cell stimulation assays we could unambiguously distinguish between unvaccinated and vaccinated animals.

## Results

### Generation of the ZIKV RNA nanoparticle vaccine

**Figure 1:**
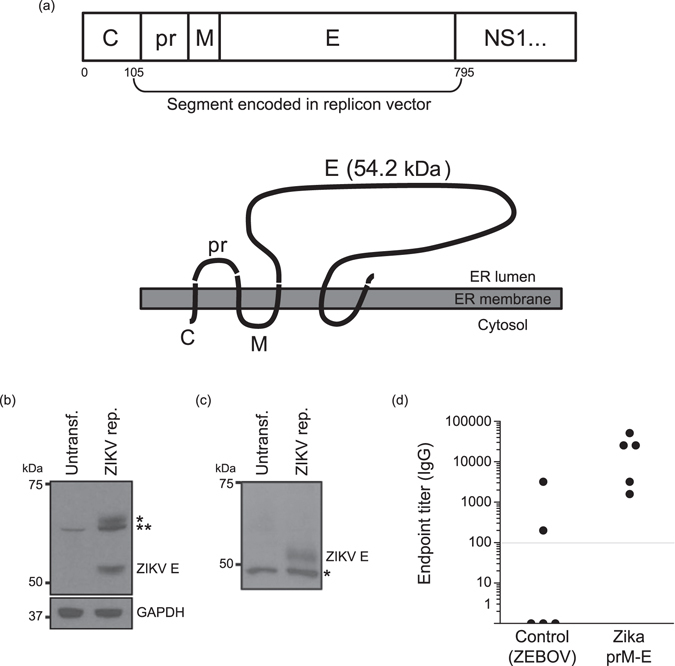
RNA nanoparticle vaccine design and function. (**a**) ZIKV polypeptide region encoded in the RNA replicon as the target antigen (numbers refer to amino acid positions), and predicted topology of the expressed truncated protein segment. (**b**) Immunoblot performed on lysates of replicon-transfected BHK21 cells 72 h post-transfection with an anti-ZIKV E rabbit polyclonal antibody. Asterisks indicate additional high-molecular weight bands possibly corresponding to incompletely processed E protein (*) or a combination of incompletely processed E protein and background band also present in untransfected control cells (**). (**c**) Immunoblot performed as in (**b**) but on conditioned supernatant removed from transfected cells. The asterisk indicates a common background band presumably produced by cross-reactivity of the polyclonal antibody used for detection against a culture medium serum component. The membrane was cropped above the 75 kDa marker to eliminate contaminating bovine serum albumin (from the culture medium) bands from the blot. (**d**) Anti-ZIKV recombinant E protein IgG titers from C57BL/6 mice immunized with the indicated RNA nanoparticle vaccine.

The premembrane (prM) and envelope (E) proteins of ZIKV isolate Z1106033 (derived from an Asian lineage virus, isolated from a patient in Suriname at the onset of the late-2015 expansion of the virus in the Americas)^29^ were encoded as a single open reading frame into an RNA replicon vector (Fig. 1a). RNA was transcribed from the plasmid *in vitro*, and expression of the correct post-translationally cleaved envelope glycoprotein was confirmed by immunoblot in transfected hamster kidney cells (BHK21) after 3 days using a polyclonal antiserum against ZIKV E protein. Expression of the expected ~54 kDa protein product was observed (Fig. 1b). Additional bands (indicated by asterisks) were detected that may correspond to incompletely processed ZIKV E protein. The full-length prM-E polyprotein segment encoded in the replicon has a predicted molecular weight of 73.4 kDa after signal peptidase processing to release the prM N-terminus. Incomplete signal peptidase cleavage of the M-E junction would yield a 62 kDa intermediate polypeptide. As alphaviral replicons saturate and substantially disrupt the secretory pathway in BHK21 cells over time, retention of polyprotein processing intermediates in transfected cells is not surprising^30, 31^. Regardless, only the correctly processed ~54 kDa polypeptide was observed to accumulate in the culture medium conditioned by the transfected cells, indicating that antigen release took place (Fig. 1c), as expected: expression of the prM and E proteins of flaviviruses results in the release of sub-viral particles (SVPs) of slightly smaller size than capsid-containing virons^32^. As SVPs are antigenically similar to functional virions, their production underlies the performance of nucleic acid-based vaccines. The RNA replicon was formulated with a modified dendrimer nanomaterial to form MDNP vaccines, as described^28^ (Figure S1). C57BL/6 mice (n = 5) were immunized with this formulation by intramuscular (i.m.) injection. A control group was immunized with a similar RNA replicon vaccine encoding the Zaire Ebola virus glycoprotein (ZEBOV). After immunization, serum was collected and IgG reactivity against a recombinant ZIKV envelope protein was determined by ELISA (Fig. 1d). All immunized mice exhibited IgG reactivity against the ZIKV envelope protein, while only two of the control mice were seropositive above the detection limit of the assay. These two seropositives may have been the result of nonspecific cross-reactivity due to structural similarities between flavivirus and filovirus envelope proteins^33–36^. Nevertheless, the subsequently identified Class I MHC-restricted epitope distinguished between the two immunized groups unambiguously in a cytokine release assay (described below).

### Peptide screening for CD8^+^ envelope gene-derived epitopes

**Figure 2:**
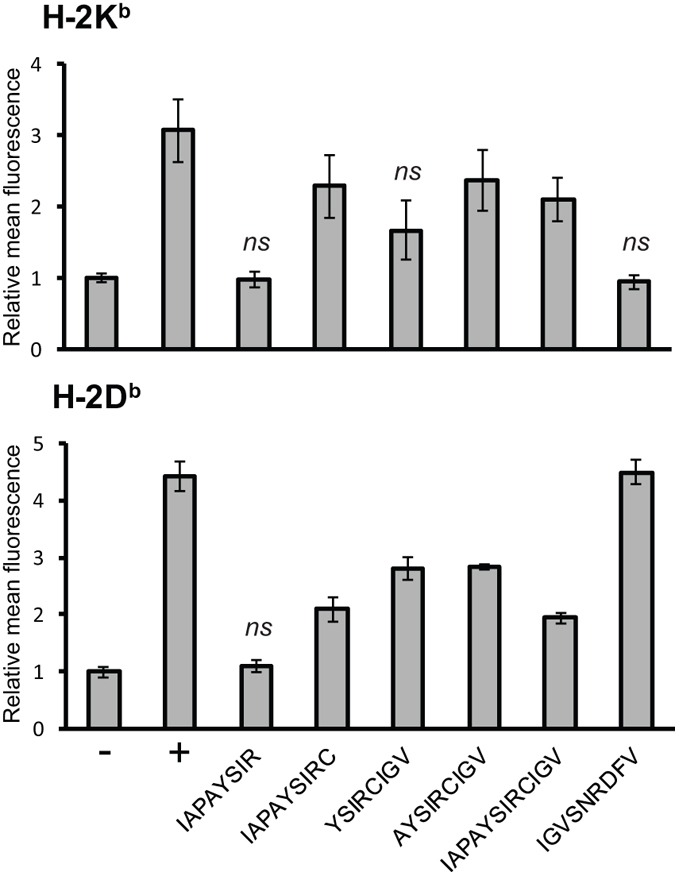
RMA-S cell peptide-binding assay. The binding of the indicated peptides to MHC class I molecules was determined by surface stabilization of H-2Kb or H-2Db molecules on RMA-S cells as measured by flow cytometry. Assays were performed in triplicate. The “−” denotes that no peptide was added, and the “+” denotes the addition of a peptide known to bind to the measured MHC class I molecule (OVA SIINFEKL for H-2K^b^, and HPV16 E7 RAHYNIVTF for H-2D^b^). Fluorescence intensities were normalized to the no peptide control and data are shown as the means ± SD. *ns*, not statistically significant from the no-peptide control value according to an unpaired two-tailed t-test (*p* > 0.05).

An overlapping 15-mer peptide library (Table S1) spanning amino acids 105 to 713 of the ZIKV polyprotein (Fig. 1a) was generated, and individual pools of 5–8 peptides each were used in *ex vivo* stimulation experiments on splenocytes isolated from an IgG-positive ZIKV-immunized mouse. Four pools (pools #5, 6, 7, 11) were identified that induced interferon gamma (IFNγ) expression in CD8^+^ T cells (Table 1). Peptides from each of these pools were then tested individually, and 7 peptides were found to induce a response (defined as IFNγ expression in >0.2% of CD8^+^ T cells, as nearly all other peptides stimulated <0.1%; Table 1). Three stimulatory contiguous peptides from pool #6 covered a 23 amino acid span corresponding to positions 284 to 306 of the ZIKV polypeptide. Two of these three contained a distinctive H-2D^b^-compatible 9-mer sequence, based on the presence of an asparagine anchor at position 5 and a hydrophobic C terminus (valine) at position 9. H-2D^b^- and H-2K^b^-binding epitopes from all 7 individual ‘hits’ were computationally evaluated based on a prediction of the half maximal inhibitory concentration (IC_50_) using the artificial neural network (ANN) algorithm from the Immune Epitope Database Analysis Resource^37, 38^. The H-2D^b^-restricted peptide IGVSNRDFV was calculated to have an IC_50_ of approximately one order of magnitude lower than any other predicted sequence. The next best ANN-predicted epitope-containing peptide (IAPAYSIRCIGV), assigned to H-2K^b^ by the algorithm, was also selected for comparison in further experiments. The candidate peptides were prepared by solid-phase peptide synthesis (SPPS) on a flow-based peptide synthesizer^39^ using 2-chlorotrityl chloride resin and Fmoc-protected building blocks to yield peptides with free termini. In an *in vitro* Class I MHC peptide binding experiment utilizing RMA-S cells^40^, IGVSNRDFV significantly stabilized surface H-2D^b^ molecules to the same degree as the well-known H-2D^b^-restricted human papillomavirus type 16 (HPV16) E7_49–57_ epitope^41^, which served as a positive control for H-2D^b^ binding (Fig. 2). The 12-mer peptide predicted to contain H-2K^b^ epitopes indeed exhibited H-2K^b^ stabilization, as did two 9-mer and one 8-mer derivatives thereof, though not to the same degree as the well-established H-2K^b^ ovalbumin SIINFEKL peptide^42^. Interestingly, an intermediate degree of H-2D^b^ stabilization was also observed with these peptides.

**Table 1:** 15-mer Peptide candidates tested individually for CD8^+^ T cell cytokine release.

Pool #	Peptide #	Sequence	% IFNγ + CD8 + T cells	Anchor Compatible Class I H-2 allele
Pool 5	33	QTWLESREYTKHLIR	0.029	
	34	ESREYTKHLIRVENW	0.000	
	35	YTKHLIRVENWIFRN	0.140	
	36	LIRVENWIFRNPGFA	0.007	
	37	ENWIFRNPGFALAAA	0.022	
	**38**	**FRNPG** **F** **ALAAAAIAW**	**0.490**	**Kb**
	39	GFALAAAAIAWLLGS	0.021	
	40	AAAAIAWLLGSSTSQ	0.100	
Pool 6	41	IAWLLGSSTSQKVIY	0.029	
	42	LGSSTSQKVIYLVMI	0.000	
	43	TSQKVIYLVMILLIA	0.037	
	44	VIYLVMILLIAPAYS	0.053	
	45	VMILLIAPAYSIRCI	0.016	
	**46**	**LIAPA** **Y** **SIRCIGVSN**	**0.320**	**Kb**
	**47**	**AYSIRCIGVS** **N** **RDFV**	**0.440**	**Db**
	**48**	**RCIGVS** **N** **RD** **F** **VEGMS**	**0.380**	**Db/Kb**
Pool 7	49	VSNRDFVEGMSGGTW	0.170	
	50	DFVEGMSGGTWVDVV	0.045	
	51	GMSGGTWVDVVLEHG	0.027	
	52	GTWVDVVLEHGGCVT	0.018	
	53	DVVLEHGGCVTVMAQ	0.017	
	54	EHGGCVTVMAQDKPT	0.027	
	**55**	**CVTV** **M** **AQDKPTVDIE**	**0.350**	**Db**
	56	MAQDKPTVDIELVTT	0.000	
Pool 11	81	NLEYRIMLSVHGSQH	0.046	
	82	RIMLSVHGSQHSGMI	0.009	
	83	SVHGSQHSGMIVNDT	0.009	
	84	SQHSGMIVNDTGHET	0.000	
	85	GMIVNDTGHETDENR	0.027	
	86	NDTGHETDENRAKVE	0.009	
	**87**	**HETDE** **N** **RAKVEITPN**	**0.340**	**Db**
	**88**	**ENRAKVEITP** **N** **SPRA**	**0.350**	**Db**

Peptides that induced IFNγ expression in >0.2% of CD8^+^ T cells were considered hits. Possible anchoring residues are underlined.

### The H-2D^b^-restricted ZIKV envelope-derived peptide IGVSNRDFV stimulates *ex vivo* IFNγ production in CD8^+^ T cells from ZIKV-immunized H-2b mice

**Figure 3:**
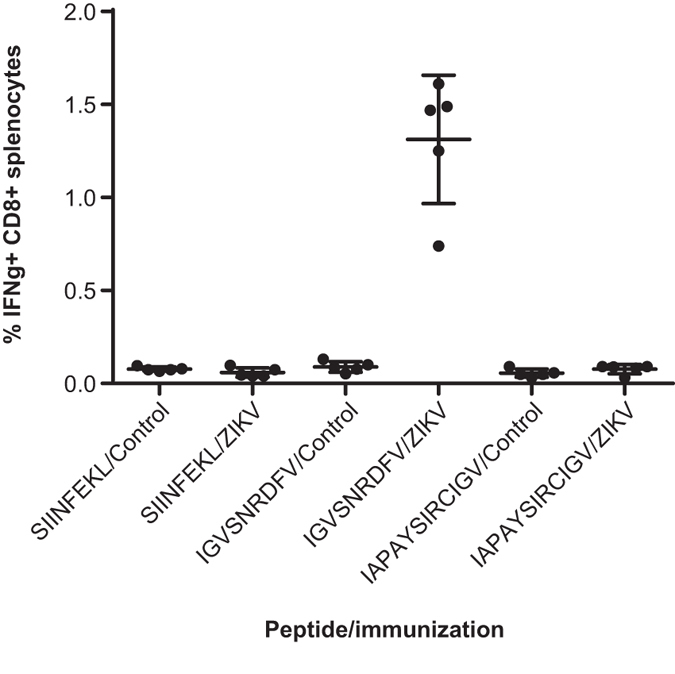
Candidate ZIKV E H-2D^b^-binding peptide stimulates CD8^+^ T cells from ZIKV-immunized mice. Splenocytes from mice vaccinated with the indicated RNA nanoparticle vaccine were stimulated *ex vivo* with 0.2 μg/mL of the indicated peptide. After 7 h, intracellular cytokine staining for IFNγ was performed and measured by flow cytometry. Error bars represent SD, and N = 5 animals per group.

Splenocytes were cultured alone or in the presence of 0.2 μg/mL of the candidate ZIKV-derived peptides or the unrelated OVA-derived H-2K^b^-restricted peptide SIINFEKL^42^. Only in ZIKV-immunized mice did we detect CD8^+^ T (~1.2-% of total) cells positive for intracellular IFNγ-staining when incubated with the candidate H-2D^b^-restricted peptide IGVSNRDFV (Fig. 3). Incubation with the putative H-2K^b^-restricted peptide IAPAYSIRCIGV resulted in no activation. This peptide is substantially longer than typical H2K^b^ epitopes; therefore, to evaluate the possibility that it failed to be correctly processed to the appropriate length *in vitro*, the additional 8-mers (IAPAYSIR and YSIRCIGV) and 9-mers (IAPAYSIRC and AYSIRCIGV) derived from this peptide tested in RMA-S binding assays were also tested separately. An additional two peptides derived from the screen ‘hits’ were selected based on the presence of anchor residues for H-2D^b^ and H-2K^b^. All peptides tested are summarized in Table 2. No peptide other than IGVSNRDFV induced a detectible CD8^+^ T cell response (Figure S2).

**Table 2:** Individual peptides tested for CD8^+^ T cell cytokine release.

Sequence	Probable Class I H-2 allele	Notes
**IGVSNRDFV**	**Db**	**Highest predicted Db-binding epitope by ANN. (IC**_**50**_ ** = 9)**
**IAPAYSIRCIGV**	**Kb**	**Highest predicted Kb-binding epitope by ANN. (IC**_**50**_ ** = 72)**
IAPAYSIRC	Kb	
IAPAYSIR	Kb	
YSIRCIGV	Kb	
AYSIRCIGV	Kb	
ETDENRAKV	Db	
RNPGFALAA	Kb	

## Discussion

Given the threat of the ZIKV outbreak and likelihood of continuing transmission worldwide, accelerated vaccine development is critical. We used a fully synthetic MDNP-based RNA replicon vaccine platform^28^ as a tool to generate a ZIKV vaccine candidate in the complete absence of reference virus stocks. We identified a Class I MHC-restricted 9-mer peptide to which a CD8^+^ T cell response was induced in immunized C57BL/6 mice, using an *ex vivo* stimulation assay. The identified IGVSNRDFV sequence is conserved across all clades of ZIKV. Our work facilitates the assessment of immune responses against the dominant ZIKV antigen without the need for recombinant production of the native glycoprotein.

This work uncovered only a single Class I MHC class I-restricted epitope from the ZIKV E protein to which a CD8^+^ T cell response was elicited in immunized mice. Given the size of the ZIKV genome and the number and size of proteins it encodes, this is somewhat surprising, and our method may have missed other, perhaps minor epitopes. However, the CD8 T cell response in C57BL/6 mice against Sendai virus provides an example of a single dominant epitope (FAPGNYPAL) being responsible for a protective CD8^+^ T cell response, despite the presence of other predicted and experimentally verified Class I MHC binders^43–45^. Mutations in H-2K^b^ that abrogate FAPGNYPAL binding make Sendai virus more pathogenic^46, 47^. In addition, a single mutation in the epitope sequence rendered the virus lethal at a dose readily handled for wild type Sendai virus by C57BL/6 mice. In this regard, it is worth pointing out that FAPGNYPAL is an excellent H-2D^b^ binder, owing to the asparagine residue at position 5^45^, but apparently no CD8^+^ T cells are induced against it in the anti-Sendai response.

A DNA ZIKV vaccine candidate is currently undergoing clinical testing^9^, but the self-limiting nature of an RNA-based vaccine possesses obvious safety advantages over DNA-based approaches, chiefly owing to a lack of potential genome integration. In the case of replicon RNAs, there is also a potential potency advantage, as self-replication in the cytoplasm can drive greater transgene expression than DNA vectors. Given the observation here that a fully synthetic MDNP-based RNA replicon nanoparticle preparation induced humoral and cellular immune responses that have been correlated with protective immunity elsewhere^24^, it will be interesting to pursue further characterization of this vaccine candidate in terms of protective potential *in vivo* in comparison to other nucleic acid and conventional protein/virus-like particle approaches. Notably, the generation of this MDNP vaccine candidate, and the elucidation of an immunodominant Class I MHC-restricted epitope, were performed without the need for ZIKV itself. This fully synthetic and chemically defined process required no more than access to the ZIKV coding sequence.

## Methods

### RNA nanoparticle vaccine production

Cloning and RNA synthesis was performed as previously described^28^. Briefly, Venezuelan equine encephalitis virus (VEEV) replicon RNAs were produced by cloning antigens into the VEEV replicon plasmid pTK126, based on the wild-type TRD strain (kindly provided by Tasuku Kitada, Weiss Laboratory, Massachusetts Institute of Technology, Cambridge, MA, USA) to replace the mVenus coding sequence located downstream of the VEEV subgenomic promoter sequence. Replicon RNAs were synthesized from these vectors after linearization with the restriction enzyme I-SceI. I-SceI cuts downstream of the VEEV 3′ untranslated region (UTR) and a short poly(A) tract of 40 base pairs (bp), and upstream of a T7 RNA polymerase promoter element preceding the VEEV 5′ UTR. RNAs were synthesized from the linearized plasmid vectors by *in vitro* transcription with MEGAscript kits (Life Technologies, Carlsbad, CA, USA), 5′ capped to produce cap-1 structured 2′-O-methylated 7-methylguanylate 5′ ends using the ScriptCap m7G Capping System and 2′-O-methyltransferase kits (CellScript Inc., Madison, WI, USA), and 3′ poly(A)-tailed using A-Plus Poly(A) Polymerase Tailing kits (CellScript) according to manufacturer protocols. MDNP formulation was performed as we described previously^28^. Nanoparticles were characterized with a Zetasizer Nano-ZS machine (Malvern, UK). The concentration of RNA was determined by theoretical mass balance calculations and confirmed by spectrophotometry (NanoDrop, Thermo Fisher Scientific, Waltham, MA, USA).

### ZIKV antigen detection by immunoblot

Antigen expression was assayed in transfected baby hamster kidney cells (BHK21) maintained at 37 °C and 5% (v/v) CO_2_ in Eagle’s minimal essential medium supplemented with 5% (v/v) FBS and 2 mM sodium pyruvate (Invitrogen, Carlsbad, CA, USA). Cells growing in log phase were transfected with the indicated RNA at 50–75% confluence using TransIT-mRNA transfection kits (Mirus Bio, Madison, WI, USA). After 72 h, the cells were lysed, and proteins were extracted in CelLytic™ M Mammalian Cell Lysis/Extraction Reagent (Sigma-Aldrich, St. Louis, Missouri) supplemented freshly with 25 U/μL benzonase (EMD Millipore, Billerica, MA, USA), and cOmplete, Mini EDTA-free Protease Inhibitors (Roche Life Science, Mannheim, Germany) according to the manufacturer’s recommendations. Subsequently, lysates were mixed with Laemmli SDS buffer (Boston Bioproducts Inc., Ashland, MA, USA) and separated by sodium dodecyl sulfate polyacrylamide gel electrophoresis (SDS-PAGE) on 4–12% gradient Bolt® Bis-Tris gels (Thermo Fisher, Waltham, MA, USA) before transfer to PVDF membranes for immunoblotting. Membranes were blocked with 10% milk in Tris-buffered saline with 0.1% Tween-20 (TBST), incubated with primary detection antibodies in blocking buffer (10% milk in TBST) for 1 h at room temperature, and then incubated with horseradish peroxidase (HRP)-conjugated secondary antibodies in blocking buffer for 1 h at room temperature. For ZIKV envelope antigen detection, membranes were incubated with a rabbit polyclonal anti-Zika primary antibody (product number GTX133314, GeneTex, Inc., Irvine, CA, USA) diluted 1:1000, followed by incubation with an anti-mouse HRP-conjugated secondary antibody diluted 1:10,000 (GE Healthcare, Pittsburgh, PA, USA). Enhanced luminol-based detection was performed using Western Lightning-ECL kits (Perkin-Elmer, Boston, MA, USA). For detection of secreted antigen, cells were transfected exactly as described, but medium was switched to reduced serum (1% FBS) 16 hrs post-transfection, and the conditioned culture medium was harvested 72 hrs post-transfection. The conditioned medium was clarified by centrifugation to remove cellular debris, mixed 1:1 with Laemmli SDS buffer, and immunoblot was performed as described above for lysates except that the membrane was cropped at the 75 kDa marker band to remove large contaminating BSA bands from the culture medium that were visible upon Ponceau S staining. Raw images of the immunoblot films without cropping or annotation are shown in Figure S3.

### Peptide synthesis

#### A) Overlapping peptide pool screening

An overlapping peptide library consisting of 15-mers with 5 amino acid overlaps was synthesized by Mimotopes Pty Ltd (Clayton, Australia), and individual lyophilized peptides were dissolved in dimethyl sulfoxide (DMSO) at a concentration of ~9 mg/mL. These peptides were screened in *ex vivo* splenocyte stimulation assays as described below.

#### B) Large scale synthesis and validation of target epitope sequences

2-Chlorotrityl chloride resin (125 mg, 1.6 mmol/g, Chemprep, Miami, FL, USA) was swollen in dichloromethane (10 mL) followed by the addition of the first amino acid (0.6 mmol) and N,N-diisopropylethylamine (DIPEA; 500 μL). After agitation at room temperature for 1 h, the solution was drained and the resin was washed three times with dimethylformamide (DMF), and capped with a mixture of dichloromethane (DCM)/MeOH/DIPEA (80:15:5 v/v/v). After 30 min, the resin was washed and transferred to the flow reaction vessel. Flow-based peptide synthesis was then performed following an established protocol^39^. Briefly, each coupling cycle involved four steps: washing (60 s), deprotection (45 s), washing (90 s), and coupling. All reactions were conducted at 60 °C. Except for coupling, all cycles were conducted at a flow rate of 20 mL/min. For the coupling step, 2 mmol (10 eq.). Fmoc-protected amino acids were dissolved in 5 mL of 0.4 M 2-(1H-benzotriazol-1-yl)-1, 1, 3, 3-tetramethyluronium hexafluorophosphate (HBTU) solution in DMF, followed by the addition of 500 μL DIPEA. The mixture was injected by a syringe pump at 60 mL/h. Deprotection was conducted by switching the flow from DMF to 20% piperidine in DMF. The finished sequence was cleaved and deprotected using TFA/TIPS/water/phenol/EDT (90:2.5:2.5:2.5:2.5, v/v/v/v/v), precipitated in cold ether, and purified by high-performance liquid chromatography (HPLC; Gemini C18 column, 5 μm, 10 × 250 mm; Phenomenex, Torrance, CA, USA). The corresponding fractions were analyzed via liquid chromatography–mass spectrometry (LC-MS; Waters Xevo system equipped with UPLC-C18 column, Manchester, UK; Figure S4), and the following masses were observed: IGVSNRDFV [M + H]^+^ calculated = 1006.523, observed = 1006.515; IAPAYSIRCIGV [M + H]^+^ calculated = 1262.693, observed = 1262.679.

### Mice and immunization

All animal studies were performed at the Whitehead Institute for Biomedical Research, Cambridge, MA, and were in accordance with protocols approved by the Institutional Animal Care and Use Committee (IACUC) to comply with all applicable local, state, and federal regulations. Female C57BL/6 mice were primed on day 0 and boosted 5 weeks later with 40 µg doses (based on RNA mass) of the nanoparticle vaccine by bilateral i.m. injection as previously described^28^. Blood was collected 4 weeks after the boost, and splenocytes were isolated and cryogenically stored in fetal bovine serum (FBS) with 10% DMSO before analysis.

### Serum ELISA

High binding surface-treated polystyrene 96-well microplates (Corning, Corning, NY, USA) were coated overnight at 4 °C with 0.5 µg/mL recombinant Zika E protein (MyBioSource Inc., San Diego, CA, USA) in 100 mM carbonate/bicarbonate buffer (pH 9). Plates were blocked for 2 hours with blocking buffer (PBS with 10% FBS, Life Technologies) at room temperature, and serum was applied to wells in duplicate at a minimum 1:100 dilution in blocking buffer, and incubated for 2 hours at room temperature. Plates were washed with wash buffer (PBS with 0.05% Tween-20), and incubated at room temperature with anti-mouse IgG-HRP (GE Healthcare) diluted 1:3000 in blocking buffer for 1 h. After 4 washes with wash buffer, the plates were developed with tetramethylbenzidine (TMB) substrate (Sigma, St. Louis, MO, USA) for 20–30 min, and the reaction was stopped by the addition of one volume of 1 M HCl before measuring absorbance at 450 nm. Endpoint titers were determined using an optical density (OD) 450 nm cutoff of 0.08.

### *Ex vivo* splenocyte stimulation assay

The peptides were commercially provided in a 96-well format, and were pooled in equivalent volumes by combining individual columns for *ex vivo* splenocyte stimulation assays, which resulted in a final pooled concentration of ~1 μg/mL in each culture. After the identification of four pools that stimulated enhanced IFNγ expression in CD8^+^ T cells, individual peptides were tested in identical stimulation assays at ~4 μg/mL. A total of 10^7^ splenocytes in 100 μL of complete media (RPMI 1620 supplemented with GlutaMAX, 8% FBS, 1 mM nonessential amino acids, 1 mM sodium pyruvate, 10 mM HEPES, penicillin/streptomycin, Life Technologies; and 50 μM 2-mercaptoethanol, Sigma) were added to each well of a 96-well flat-bottom cell culture plate. After a 1-h pre-incubation, the cells were incubated with IL-2 (10 U/mL), anti-CD28, anti-CD49d (0.5 μg/mL each, BioLegend), and GolgiStop (BD Biosciences) diluted 1:1500. For evaluation of the synthetic H-2D^b^ and H-2K^b^ candidate peptides and the SIINFEKL control peptide, IL-2 and anti-CD28/CD49d were omitted, as costimulatory signals proved unnecessary for IFNγ induction. Intracellular cytokine staining for IFNγ^+^CD8^+^ T cells was performed as described previously^28^, and populations were analyzed on a BD LSR II Flow Cytometer (BD Biosciences). In the screening, ‘hits’ were defined if the percentage of IFNγ^+^CD8^+^ T cells was >0.2% of total CD8^+^ T cells (background levels in control cultures were consistently <0.2%). A minimum of 10000 CD8^+^ T cells were analyzed per sample.

### Peptide-binding assay with RMA-S cells

MHC class I molecules (H-2K^b^ and H-2D^b^) lacking peptides are expressed on the surface of RMA-S cells upon culture at reduced temperature^40^. These cell surface empty class I molecules are thermolabile at 37 °C, but are stabilized by the addition of MHC binding peptides. RMA-S cells were cultured at 26 °C for 18 h and washed with PBS. Then, 10^5^ cells/well were seeded onto a 96-well cell culture plate. ZIKV or control peptides (OVA_257–264_ for H-2K^b^ and HPV16 E7_49–57_ for H-2D^b^) were added to each well at 30 μg/10^6^ cells. The cells were then incubated at 37 °C for 2 h and washed twice with ice cold PBS. For H-2K^b^ detection, the cells were stained with a mouse FITC-conjugated anti-H-2K^b^ antibody (1:200 dilution in PBS with 1% BSA, Biolegend) for 45 min at 4 °C. For H-2D^b^ detection, the cells were stained with a mouse anti-H-2D^b^ antibody (1:500 dilution in PBS with 1% BSA, BD Biosciences) for 45 min, followed by incubation with a goat anti-mouse Alexa Fluor 488-conjugated secondary antibody (1:10000 dilution in PBS with 1% BSA, Thermo Fisher) for 30 min at 4 °C. The cells were then washed three times with ice cold PBS, and fluorescence intensity was measured with a BD Accuri C6 Flow Cytometer (BD Biosciences) and normalized to the no peptide control.

## Electronic supplementary material

Supplementary Information for
