# Supplementary material for: An RNA nanoparticle vaccine against Zika virus elicits antibody and CD8+ T cell responses in a mouse model
Source: Sci Rep. 2017 Mar 21;7:252. doi: 10.1038/s41598-017-00193-w (PMC5427874; doi:10.1038/s41598-017-00193-w)
Supplement: Supplementary file 1 — Supplementary Information for [file 41598_2017_193_MOESM1_ESM.pdf]

Supplementary Information for

An RNA nanoparticle vaccine against Zika virus elicits antibody and CD8+ T cell responses in a mouse model

Jasdave S. Chahal[1,2], Tao Fang[1], Andrew W. Woodham[1], Omar F. Khan[2], Jingjing Ling[1], Daniel G. Anderson[2] and Hidde L. Ploegh[1]

[1]Whitehead Institute for Biomedical Research, 9 Cambridge Center, Cambridge, MA, 02142, USA.

[2]The Koch Institute for Integrative Cancer Research, Massachusetts Institute of Technology, Cambridge, MA, 02142, USA.

Table S1. Peptide pools tested in initial screen for CD8-stimulating epitopes.

Pool #	Peptide #	Sequence
Pool 1	1	MGADTSVGIVGLLLT
	2	TSVGIVGLLLTTAMA
	3	IVGLLLTTAMAAEVT
	4	LLTTAMAAEVTRRGS
	5	AMAAEVTRRGSAYYM
	6	EVTRRGSAYYMYLDR
	7	RGSAYYMYLDRNDAG
	8	YYMYLDRNDAGEAIS
Pool 2	9	LDRNDAGEAISFPTT
	10	DAGEAISFPTTLGMN
	11	AISFPTTLGMNKCYI
	12	PTTLGMNKCYIQIMD
	13	GMNKCYIQIMDLGHT
	14	CYIQIMDLGHTCDAT
	15	IMDLGHTCDATMSYE
	16	GHTCDATMSYECPML
Pool 3	17	DATMSYECPMLDEGV
	18	SYECPMLDEGVEPDD
	19	PMLDEGVEPDDVDCW
	20	EGVEPDDVDCWCNTT
	21	PDDVDCWCNTTSTWV
	22	DCWCNTTSTWVVYGT
	23	NTTSTWVVYGTCHHK
	24	TWVVYGTCHHKKGEA
Pool 4	25	YGTCHHKKGEARRSR
	26	HHKKGEARRSRRAVT
	27	GEARRSRRAVTLPSH
	28	RSRRAVTLPSHSTRK
	29	AVTLPSHSTRKLQTR
	30	PSHSTRKLQTRSQTW
	31	TRKLQTRSQTWLESR
	32	QTRSQTWLESREYTK
Pool 5	33	QTWLESREYTKHLIR
	34	ESREYTKHLIRVENW
	35	YTKHLIRVENWIFRN

	36	LIRVENWIFRNPGFA
	37	ENWIFRNPGFALAAA
	38	FRNPGFALAAAAIAW
	39	GFALAAAAIAWLLGS
	40	AAAAIAWLLGSSTSQ
Pool 6	41	IAWLLGSSTSQKVIY
	42	LGSSTSQKVIYLVMI
	43	TSQKVIYLVMILLIA
	44	VIYLVMILLIAPAYS
	45	VMILLIAPAYSIRCI
	46	LIAPAYSIRCIGVSN
	47	AYSIRCIGVSNRDFV
	48	RCIGVSNRDFVEGMS
Pool 7	49	VSNRDFVEGMSGGTW
	50	DFVEGMSGGTWVDVV
	51	GMSGGTWVDVVLEHG
	52	GTWVDVVLEHGGCVT
	53	DVVLEHGGCVTVMAQ
	54	EHGGCVTVMAQDKPT
	55	CVTVMAQDKPTVDIE
	56	MAQDKPTVDIELVTT
Pool 8	57	KPTVDIELVTTTVSN
	58	DIELVTTTVSNMAEV
	59	VTTTVSNMAEVRSYC
	60	VSNMAEVRSYCYEAS
	61	AEVRSYCYEASISDM
	62	SYCYEASISDMASDS
	63	EASISDMASDSRCPT
	64	SDMASDSRCPTQGEA
Pool 9	65	SDSRCPTQGEAYLDK
	66	CPTQGEAYLDKQSDT
	67	GEAYLDKQSDTQYVC
	68	LDKQSDTQYVCKRTL
	69	SDTQYVCKRTLVDRG
	70	YVCKRTLVDRGWGNG
	71	RTLVDRGWGNGCGLF
	72	DRGWGNGCGLFGKGS
Pool 10	73	GNGCGLFGKGSLVTC
	74	GLFGKGSLVTCAKFA
	75	KGSLVTCAKFACSKK
	76	VTCAKFACSKKMTGK
	77	KFACSKKMTGKSIQP
	78	SKKMTGKSIQPENLE
	79	TGKSIQPENLEYRIM
	80	IQPENLEYRIMLSVH
Pool 11	81	NLEYRIMLSVHGSQH
	82	RIMLSVHGSQHSGMI
	83	SVHGSQHSGMIVNDT
	84	SQHSGMIVNDTGHET
	85	GMIVNDTGHETDENR
	86	NDTGHETDENRAKVE
	87	HETDENRAKVEITPN
	88	ENRAKVEITPNSPRA
Pool 12	89	KVEITPNSPRAEATL
	90	TPNSPRAEATLGGFG
	91	PRAEATLGGFGSLGL

	92	ATLGGFGSLGLDCEP
	93	GFGSLGLDCEPRTGL
	94	LGLDCEPRTGLDFSD
Pool 13	95	CEPRTGLDFSDLYYL
	96	TGLDFSDLYYLTMNN
	97	FSDLYYLTMNNKHWL
	98	YYLTMNNKHWLVHKE
	99	MNNKHWLVHKEWFHD
	100	HWLVHKEWFHDIPLP
	101	HKEWFHDIPLPWHAG
	102	FHDIPLPWHAGADTG
Pool 14	103	PLPWHAGADTGTPHW
	104	HAGADTGTPHWNNKE
	105	DTGTPHWNNKEALVE
	106	PHWNNKEALVEFKDA
	107	NKEALVEFKDAHAKR
	108	LVEFKDAHAKRQTVV
	109	KDAHAKRQTVVVLGS
	110	AKRQTVVVLGSQEGA
Pool 15	111	TVVVLGSQEGAVHTA
	112	LGSQEGAVHTALAGA
	113	EGAVHTALAGALEAE
	114	HTALAGALEAEMDGA
	115	AGALEAEMDGAKGRL
	116	EAEMDGAKGRLSSGH
	117	DGAKGRLSSGHLKCR
	118	GRLSSGHLKCRLKMD
Pool 16	119	SGHLKCRLKMDKLRL
	120	KCRLKMDKLRLKGVS
	121	KMDKLRLKGVSYSLC
	122	LRLKGVSYSLCTAAF
	123	GVSYSLCTAAFTFTK
	124	SLCTAAFTFTKIPAE
	125	AAFTFTKIPAETLHG
	126	FTKIPAETLHGTVTV
Pool 17	127	PAETLHGTVTVEVQY
	128	LHGTVTVEVQYAGTD
	129	VTVEVQYAGTDGPCK
	130	VQYAGTDGPCKVPAQ
	131	GTDGPCKVPAQMAVD
	132	PCKVPAQMAVDMQTL
	133	PAQMAVDMQTLTPVG
	134	AVDMQTLTPVGRLIT
Pool 18	135	QTLTPVGRLITANPV
	136	PVGRLITANPVITES
	137	LITANPVITESTENS
	138	NPVITESTENSKMML
	139	TESTENSKMMLELDP
	140	ENSKMMLELDPPFGD
	141	MMLELDPPFGDSYIV
	142	LDPPFGDSYIVIGVG
Pool 19	143	FGDSYIVIGVGEKKI
	144	YIVIGVGEKKITHHW
	145	GVGEKKITHHWHRSG
	146	KKITHHWHRSGSTIG
	147	HHWHRSGSTIGKAFE

148 RSGSTIGKAFEATVR
149 TIGKAFEATVRGAKR
150 AFEATVRGAKRMAVL

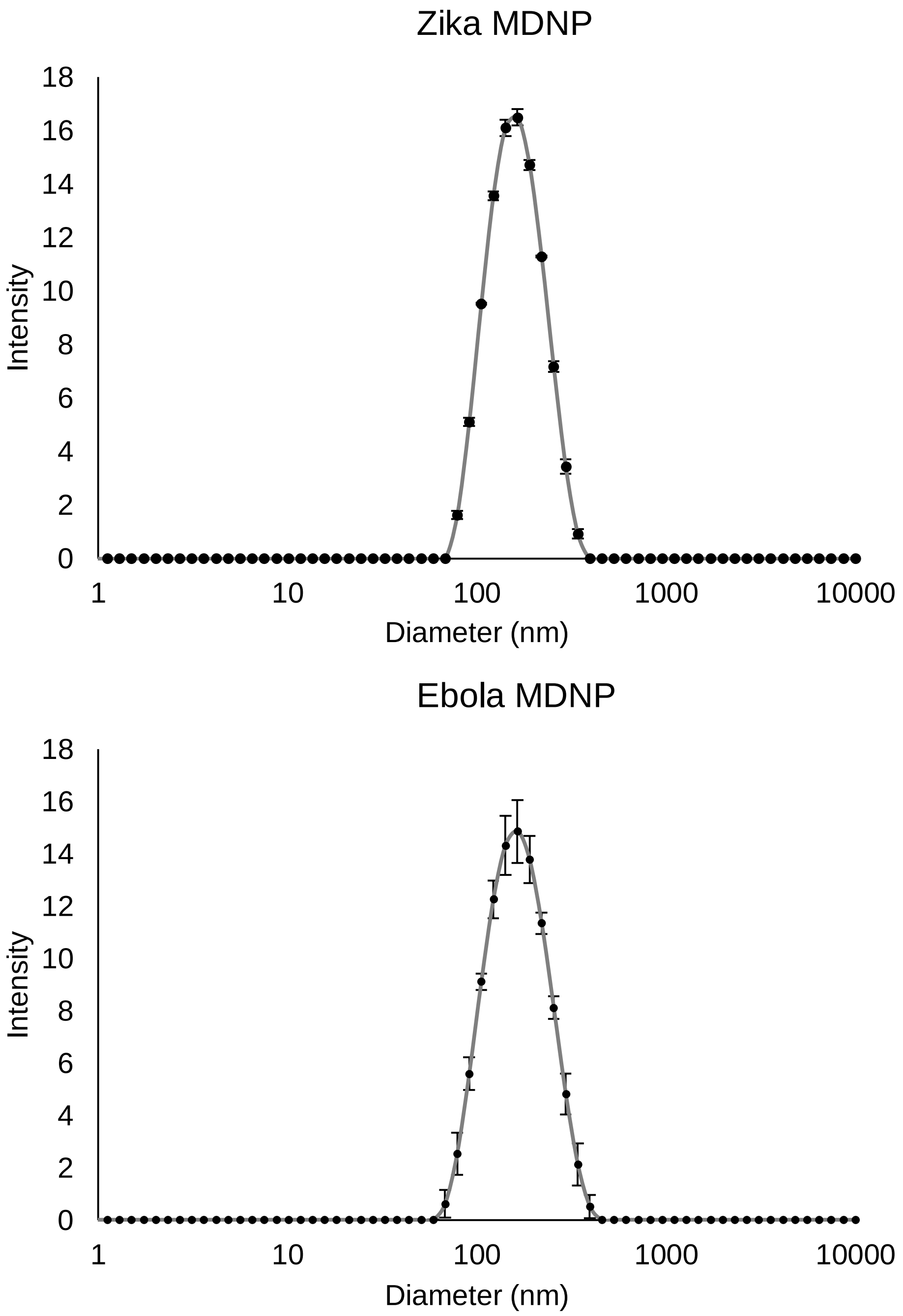

Figure S1. Modified dendrimer nanoparticle (MDNP) size distribution. MDNP vaccines suspended in PBS were characterized by dynamic light scattering and the diameters by intensity are shown. N = 3 and error bars represent SD.

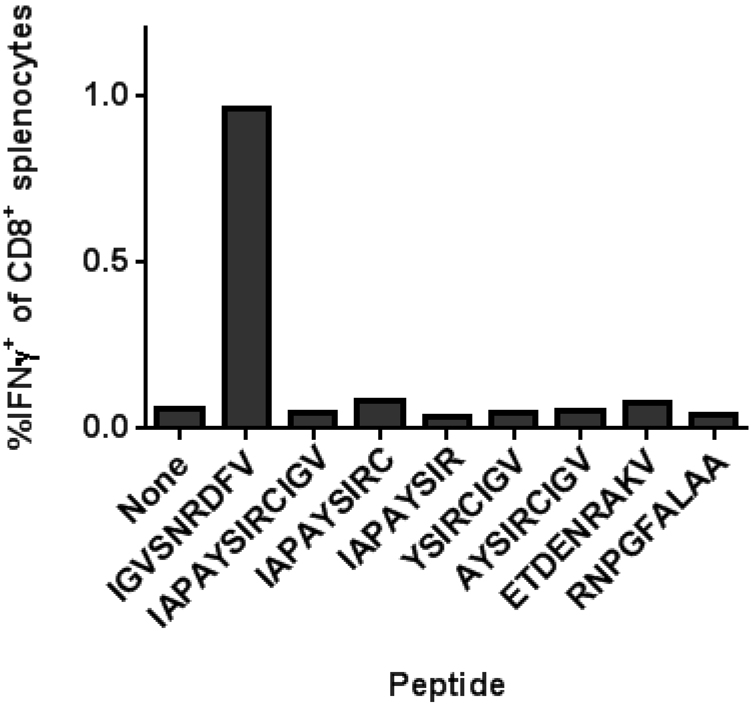

Figure S2. Screening of additional ZIKV E protein-derived peptides for activation of CD8+ T cells from a single ZIKV-immunized mouse. Splenocytes from a mouse vaccinated with RNA nanoparticle vaccine against the ZIKV E gene were stimulated *ex vivo* with 0.2 μg/ml of the indicated peptide. After 7 hrs. of culture, intracellular cytokine staining for IFNγ was performed.

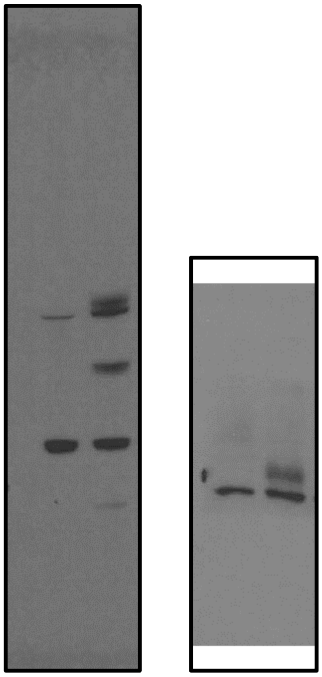

Figure S3. Raw image data for immunoblots for figures 1b (left) and 1c (right). Original, unaltered scans of films developed after chemiluminescence reactions of the indicated blots, with entire membrane lengths shown.

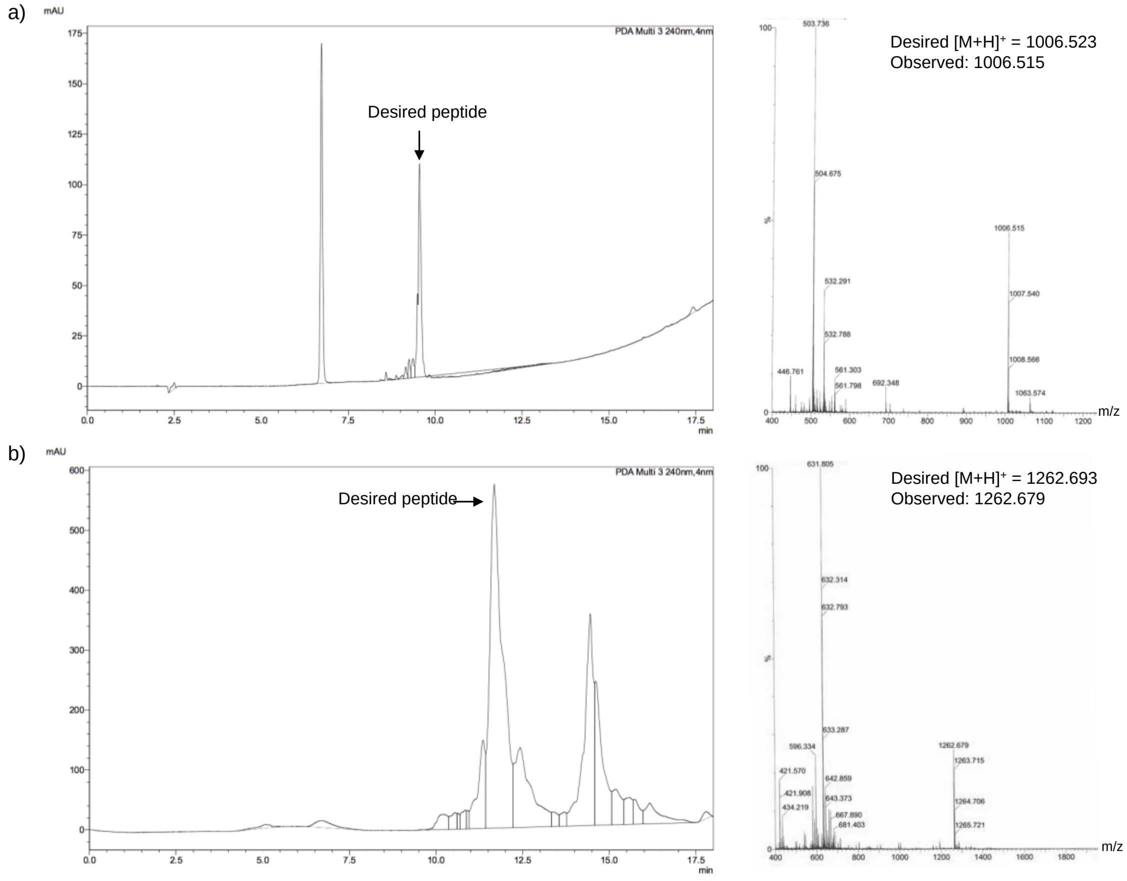

Figure S4. Representative HPLC and LC-MS characterization of ZIKV-related peptides synthesized to validate the T cell epitopes IGVSNRDFV and IPAYSIRCIGV. (a) HPLC trace and mass of desired peak corresponding to IGVSNRDFV. (b) HPLC trace and mass of desired peak corresponding to IPAYSIRCIGV.
